# Multi-omics integrated analysis identifies causal risk factors and therapeutic targets for diabetic retinopathy

**DOI:** 10.1186/s12967-025-07353-x

**Published:** 2025-12-03

**Authors:** Jing Xu, Shuntai Chen, Mei Sun, Xi Chen, Zhenzhen Gu, Yige Zhang, Like Xie, Xiaofeng Hao

**Affiliations:** 1 Eye Hospital, China Academy of Chinese Medical Sciences, Beijing, 100040 China; 2 Postdoctoral Research Station of China Academy of Chinese Medical Sciences, Beijing, 100700 China; 3 Guang’ anmen Hospital, China Academy of Chinese Medical Sciences, Beijing, 100053 China; 4 Department of Fundus Diseases and Ophthalmic Surgery, Eye Hospital, China Academy of Chinese Medical Sciences, 33 Lugu Road, Shijingshan District, Beijing, 100040 China

**Keywords:** Diabetic retinopathy, Mendelian randomization, Transcriptomics, Risk factors, Mediation effect, Biomarkers

## Abstract

**Background:**

Diabetic retinopathy (DR) is the main cause of blindness worldwide, and its prevalence rate is constantly rising. More in-depth exploration of its risk factors and pathogenic mechanisms is needed.

**Methods:**

This study systematically identified potential therapeutic targets for DR by evaluating causal effects of 16,989 genes and 2,923 proteins on DR/subtypes via two-sample Mendelian randomization (MR), validated with colocalization/Summary-data-based Mendelian randomization (SMR). National Health and Nutrition Examination Survey (NHANES) 1999–2010 cross-sectional data (weighted logistic/Restricted cubic spline (RCS)) pinpointed key risk factors; MR explored their links to DR subtypes. Bioinformatics (bulk and single-cell transcriptomics) analyzed molecular mechanisms of shared targets (gene expression, immune infiltration, pathway enrichment). Machine learning selected key targets for models. Finally, two-step mediation MR examined how targets regulate DR via risk factors.

**Results:**

This study identified 64 core targets with causal links to DR. Subtype analysis revealed 2,128 causal genes and subtype-specific targets (e.g. 52 for background DR, 66 for proliferative DR). SMR validated these findings. NHANES data highlighted body mass index (BMI), stroke, hypertension (HBP), and C-reactive protein (CRP) as key DR risk factors, confirmed by MR. Transcriptomics identified 29 differentially expressed genes associated with both risk factors and DR, linked to immune cell regulation. Machine learning selected core targets (*LY9*, *WWP2*, etc.) and built a nomogram for DR risk prediction. Functional enrichment showed these targets enriched in chemokine/cytokine and immune-inflammatory pathways. Two-step mediation MR further revealed *LY9, ARHGAP1,* and *WWP2* influence DR subtypes via regulating BMI, CRP, and HBP.

**Conclusion:**

This study systematically elucidates the key risk factors, potential molecular mechanisms, and core regulatory targets of DR through multi-omics integration, causal inference, and bioinformatics approaches. The results indicate that inflammation, immune dysregulation, and metabolic disorders play crucial roles in the pathogenesis of DR. Key genes such as *LY9, ARHGAP1,* and *WWP2* could serve as potential intervention targets, offering theoretical foundations and strategic support for early warning and precision treatment of DR.

**Supplementary information:**

The online version contains supplementary material available at 10.1186/s12967-025-07353-x.

## Introduction

Diabetic retinopathy (DR) is a retinal vascular disease caused by diabetes mellitus (DM), characterized by changes in the retinal microvasculature. It can lead to visual impairment and even blindness, making it one of the most common microvascular complications of diabetes [1]. Studies on the disease burden of DR show that in 2020, DR affected 103 million people worldwide, with 1.07 million individuals losing their vision due to DR, accounting for 2.5% of global blindness and 1.9% of moderate to severe visual impairment [2]. As the incidence of DM continues to rise, the disease burden of DR is expected to increase further, making DR a critical global public health issue [3].

Currently, the main treatments for DR include laser photocoagulation, anti-VEGF therapy, and corticosteroid vitreous injections. Although these treatments have achieved satisfactory clinical results, they have certain limitations, mainly reflected in the high treatment costs and the potential risks associated with invasive drug administration [4]. Therefore, the search for early, safe, and effective disease prevention and treatment methods is of significant clinical importance. The pathogenesis of DR involves multiple pathological mechanisms, including oxidative stress, abnormalities in polyol metabolism, the abnormal accumulation of advanced glycation end products, impaired protein kinase C signaling pathways, and inflammation [5]. Changes in certain biomarkers often precede clinical symptom changes. Hence, exploring highly sensitive, specific, and accurate biomarkers is crucial for the clinical precision medicine, individualized prevention, and intervention of DR.

Mendelian randomization (MR) is a method that uses genetic variation as an instrument to study the causal relationship between exposure factors and disease outcomes, with genome-wide association studies (GWAS) databases serving as the platform for analysis [6]. Given that genetic variation is randomly assigned and constant, MR research offers the advantage of being unaffected by confounding factors and reverse causality. In 2021, the eQTLGen consortium published a study on blood-derived expression data from 31,684 individuals, identifying 16,989 cis-expression quantitative trait loci (cis-eQTL) genes [7]; in 2023, a study published in Nature analyzed plasma proteomic features of 54,219 participants from the UK Biobank [8]. This study conducted a comprehensive cis-protein quantitative trait locus (cis-pQTL) mapping of 2,923 proteins, identifying 14,287 major genetic associations. These two studies help clarify the biological mechanisms behind proteomic-genomic discoveries and provide opportunities for the development of biomarkers, predictive models, and therapeutic targets for DR.

To systematically study the causal pathways and potential targets of human genes and proteins associated with DR, we conducted a two-sample MR analysis to assess the causal effects of 16,989 genes and 2,923 proteins on DR. Summary-data-based Mendelian randomization (SMR) analysis was then used to validate these results. Further, we analyzed clinical common risk factors and their associations with DR using cross-sectional data from the National Health and Nutrition Examination Survey (NHANES) and GWAS datasets, in order to identify significant disease risk factors. Subsequently, we analyzed the related targets of these significant DR risk factors and utilized bulk and single-cell transcriptomic data to explore the expression, immune infiltration, and pathway mechanisms of common targets between the risk factors and DR. Positive targets were selected for mediation analysis to reveal how targets affect DR through risk factors, thus elucidating the potential causal mechanisms. Through this systematic study, we aim to identify potential prevention and therapeutic targets for DR.

## Study design

**Fig. 1:**
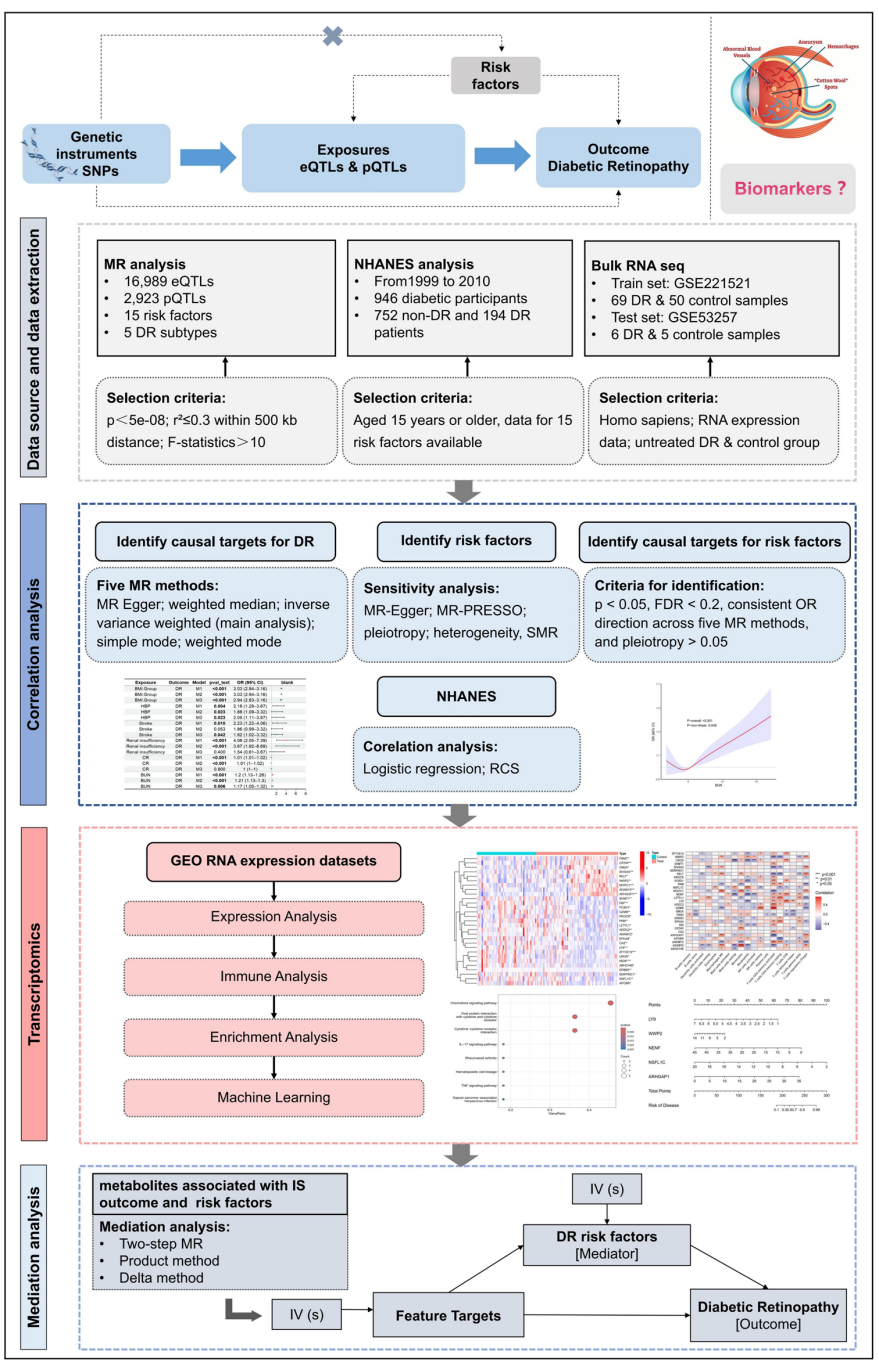
Flow chart of the study design

This study employs three methods: cross-sectional data analysis, systematic MR analysis, and transcriptomic analysis. First, we conducted a two-sample MR analysis using 16,989 cis-eQTLs and 2,923 cis-pQTLs with DR subtypes to identify genes and proteins that regulate DR, and selected DR-related targets at the multi-omics level. We then performed SMR analysis to assess the robustness of the DR-related targets. Next, using cross-sectional data from NHANES, we screened risk factors for DR and identified those significantly associated with DR. MR was used to establish causal relationships between these risk factors and DR subtypes, refining and supplementing the cross-sectional study results. The next step involved using bulk and single-cell transcriptomic data from the multi-center gene expression omnibus (GEO) human DR cohorts to analyze expression levels, immune cell infiltration, and pathway mechanisms of common targets regulating both risk factors and DR at the gene and protein levels, followed by the construction of machine learning models to further identify key feature genes associated with risk factors and DR, and validating the results in a validation cohort. Finally, we conducted two-step MR mediation analysis to examine how feature genes influence DR by regulating risk factors. The research workflow is shown in Fig. 1.

## Data source

The cross-sectional data was sourced from the NHANES (https://wwwn.cdc.gov/nchs/nhanes/Default.aspx). NHANES is a complex, multi-stage, stratified sampling health survey conducted in the United States. The data sources include structured interviews, telephone follow-ups, health checks at mobile examination centers, and laboratory sample analyses. Prior to data collection, approval was obtained from the Institutional Review Board of the National Center for Health Statistics, and participants provided written informed consent upon enrollment [9]. In this study, 946 participants from 1999 to 2010 were selected for analysis. Genetic variant data for gene expression was sourced from a study by the eQTLGen Consortium (https://www.eqtlgen.org/cis-eqtls.html) [7], which performed a meta-analysis of blood samples from 31,684 individuals, resulting in the identification of 16,989 cis-eQTLs. Protein expression genetic variant data was obtained from the UK Biobank Pharma Proteomics Project (UKB-PPP) [8], which conducted proteomic analysis on plasma samples from 54,219 participants and mapped pQTLs for 2,923 unique proteins. The pQTL data for plasma proteins is available from (https://www.synapse.org/#!Synapse:syn51365301).

The GWAS data for risk factors was sourced from the GWAS Catalog database (https://www.ebi.ac.uk/gwas/downloads/summary-statistics). Through reviewing relevant studies, we extracted the following risk factors: Body mass index (BMI), smoking, alcohol consumption, hypertension (HBP) (systolic blood pressure (SBP), diastolic blood pressure (DBP)), coronary heart disease (CHD), renal insufficiency, glucose (GLU), high-density lipoprotein cholesterol (HDL-c), low-density lipoprotein cholesterol (LDL-c), triglycerides (TG), total cholesterol (TC), blood urea nitrogen (BUN), and serum creatinine (Cr). The GWAS data for outcome events, including DR and its subtypes, was obtained from the Finnish database FinnGen (https://www.finngen.fi/fi), R9 data. This dataset includes five DR subtypes: standard DR (DR), diabetic background retinopathy (DR-BKG), severe diabetic background retinopathy (DR-BKG-SEVERE), proliferative DR (DR-PROLIF), and non-annotated DR (DR-NAS), with no sample overlap with the exposure data.

The transcriptomic data for DR was sourced from the GEO database (https://www.ncbi.nlm.nih.gov/geo/). We conducted a data search using the keyword “Diabetic Retinopathy,” with the search date being February 12, 2025. To ensure the quality and relevance of the data, we set the following inclusion criteria: 1) data types include microarray expression profiles, RT-PCR, or high-throughput sequencing; 2) species are human (Homo sapiens); 3) participants were diagnosed with DR in the original study; 4) sample sources are retina or peripheral blood; 5) sample content includes RNA expression data from untreated DR and control groups.

Detailed information about the data sources can be found in Supplementary File 1, Table S1.

## Method

### Two-sample MR-based genomic and proteomic DR target screening

We first extracted 16,989 eQTLs for genes and 2,923 pQTLs for proteins from the eQTLGen Consortium and UKB-PPP studies, respectively. Tool variables were selected based on the following criteria: 1) Single nucleotide polymorphisms (SNPs) within ±1 Mb of the gene region (cis-eQTLs and cis-pQTLs); 2) high genetic correlation with genes and proteins (*p* < 5 × 10^−8^; linkage disequilibrium (LD) r^2^ < 0.3, with a clumping window of 500 kb) [10]; to exclude weak instrumental variables that could affect causal inference, we also removed SNPs with an F-statistic less than 10. After screening, 15,711 genes and 2,597 proteins were included in the analysis. Cochran’s Q statistic was used to detect heterogeneity, and if heterogeneity between instrumental variables was detected, a random-effects model was applied; otherwise, a fixed-effects model was used. We employed five estimation models—inverse-variance weighted (IVW), weighted median, MR-Egger, Wald ratio, and simple median—to infer causal relationships between genes, proteins, and DR (across five subtypes). Due to the large number of SNPs and significant heterogeneity, the random-effects IVW model was considered the primary analytical method [11]. We also performed MR pleiotropy residual sum and outlier (MR-PRESSO) and MR-Egger tests for pleiotropy. genes and proteins associated with DR were selected using false discovery rate (FDR)-corrected *p* < 0.2 and pleiotropy > 0.05. If both gene and protein levels were significantly associated with DR, the target was considered a DR-related target.

### SMR analysis of eQtls and DR

To further assess the robustness of the DR targets identified in the MR analysis, we used the SMR analysis software provided by Yang Lab to detect pleiotropic associations between the expression levels of 16,989 gene targets and DR using the SMR method [12]. Subsequently, we applied the heterogeneity in dependent instruments (HEIDI) method to check whether the associations identified by the SMR test were driven by pleiotropy. We conducted the SMR analysis using version 1.03 of the SMR software (https://cnsgenomics.com/software/smr/#Overview) with the default parameters recommended by the developers. A *p*-value less than 0.05 was considered statistically significant.

### Analysis of risk factors and their associations with DR based on NHANES cross-sectional data

We extracted participants aged 15 years or older from the 1999–2010 cross-sectional survey data of the NHANES. DR outcomes were determined based on the questionnaire (DIQ080 - Diabetes affected eyes/had retinopathy), where a response of “Yes” indicated a DR patient, and “No” indicated a non-DR patient. Risk factors were identified according to relevant guidelines [13], including: blood glucose, glycated hemoglobin, hypertension (HBP, SBP, DBP), dyslipidemia (TG, TC, HDL-c, LDL-c), smoking, alcohol consumption, BMI, renal dysfunction (renal insufficiency, Cr, BUN), and others. In addition to demographic covariates (age, gender, race, education level, and poverty status), we also included diabetes duration and glycemic control (including the use of insulin and oral hypoglycemic agents) as important covariates in the analysis.

### Two-sample MR analysis of risk factors and DR, and screening of risk factor targets

We conducted further causal inference of the risk factors involved in the NHANES analysis and their association with DR. We selected SNPs strongly correlated with exposure factors as instrumental variables by screening GWAS summary data for exposure factors with a *p*-value < 5e-08. SNPs were removed based on a clumping criterion of kb = 10,000 and r^2^ = 0.001, and weak instrumental variables with F-statistics < 10 were excluded. Risk factors with causal relationships to DR were selected based on the criteria of *p* < 0.05, FDR < 0.2, consistent odds ratios (OR) directions across five MR methods, and pleiotropy > 0.05. To ensure the robustness of the results, we also performed MR-Egger and MR-PRESSO pleiotropy tests and Cochran’s Q heterogeneity test. For the risk factors identified as significantly associated with DR through NHANES analysis and MR validation, we conducted MR analysis on eQTL and pQTL data using the same methods and selection criteria as in section “Two-sample MR-based genomic and proteomic DR target screening”, further screening risk factor-related targets for DR.

### Transcriptomic analysis of common targets for risk factors and DR

Gene symbol annotation correction was performed on GEO data (GSE221521, which includes 69 DR samples and 50 control group samples) to obtain the expression levels of genes corresponding to DR-related targets in each sample. These gene expression levels were analyzed for differential expression between the normal and DR groups using R packages, and the results were visualized in heatmaps. Genes that showed significant differences in expression were classified as differently expressed genes (DEGs). Then, the “cor” function was used to calculate the correlation coefficients for each DEG to determine the correlation between every pair of DEGs, and the results were visualized. To further enhance the robustness and biological relevance of our findings, we additionally analyzed these statistically significant DEGs identified in peripheral blood transcriptomes using retinal samples (GSE60436 and GSE191210, comprising 9 control samples and 6 DR samples).

### Single-cell transcriptomic validation of DEGs

To further investigate the cell-type-specific expression patterns of DEGs, we analyzed a peripheral blood single-cell RNA sequencing (scRNA-seq) cohort of DR (GSE248284, comprising 3 control samples and 3 DR samples). After quality control, normalization, and dimensionality reduction, clustering analysis was performed using the Seurat R package, and cell types were annotated based on canonical marker genes. The expression levels of DEGs were then mapped across immune cell types to assess their distribution and specificity.

### Immune cell analysis and enrichment analysis of DEGs in DR samples

Using the CIBERSORT function in R, we performed 1,000 simulations to obtain the relative abundance of immune cells for each DR sample, with a total of 1 for each immune cell type. The results were visualized using bar charts. We then conducted single-sample gene set enrichment analysis (ssGSEA) to compare the differences in immune cell content between the normal and DR groups, visualizing the differences using boxplots. Based on ssGSEA scores, the correlation between DEGs and immune cells was visualized using a clustering heatmap.

We also performed gene ontology (GO) enrichment analysis for biological processes, molecular functions, and cellular components, as well as Kyoto encyclopedia of genes and genomes (KEGG) pathway enrichment analysis for the DEGs. These analyses were conducted using the R packages “clusterProfiler” and “enrichplot,” with a significance threshold of *p* < 0.05. The results were displayed using bar charts.

### Machine learning risk models and nomogram selection and validation for DR

We constructed four predictive models using the expression data of DEGs, including random forest (RF), support vector machine (SVM), generalized linear model (GLM), and extreme gradient boosting (XGB). We defined the prediction functions and calculated the results for all four models. To select feature genes from the DEGs, we created residual inverse cumulative distribution plots, residual boxplots, and receiver operating characteristic (ROC) curves for comprehensive consideration. After selecting the best model, we used the feature genes and their expression levels in the normal and DR groups to construct a nomogram. Finally, decision curves and calibration curves were generated to evaluate the accuracy of the nomogram. For external validation, the retinal cohorts described in section “Transcriptomic analysis of common targets for risk factors and dr” were used, and the same machine learning methods were applied with ROC curves plotted to verify model accuracy.

### Two-step mediation MR analysis of targets, risk factors, and DR

To further elucidate the potential mechanism by which feature targets regulate DR through risk factors, we performed a two-step MR mediation analysis [14]. First, we explored the causal relationship between genes and risk factors using two-sample MR (beta1). Subsequently, we conducted MR analysis for the statistically significant risk factors and their association with the disease (beta2). The indirect effects mediated by risk factors (mediated effect) were obtained by multiplying β1 × β2. In this study, the total effect was captured using standard univariable MR analysis (primary MR). Finally, we used the delta method to assess the significance of the mediation effect. Additionally, the mediation effect percentage was calculated by dividing the mediation effect by the total effect.

### Statistical analysis

For the MR analysis, five methods were used, including MR Egger, weighted median, IVW, simple model, and weighted model. The primary analysis method was IVW. If a tool variable for a particular exposure contained only one SNP, the Wald ratio method was applied. To correct for multiple testing, Bonferroni correction was applied to adjust the significance level threshold for multiple analyses. To evaluate the robustness of the MR analysis results, multiple methods were used, such as Cochran’s Q statistic to detect heterogeneity in the results, with a significance level set at *p* < 0.05, indicating the presence of heterogeneity. MR Egger and MR-PRESSO methods were used to assess horizontal pleiotropy, with *p* < 0.05 indicating the presence of pleiotropy.

For the NHANES analysis, cross-sectional data spanning 12 years (1999–2010) was used, applying a multi-stage, stratified, cluster, and complex sampling design. Sample weights, stratification variables, and primary sampling units were incorporated in the analysis to determine the relationship between risk factors and DR. Categorical variables were represented by counts and weighted frequencies, with Rao-Scott χ^2^ used for analysis. For continuous variables, interquartile ranges were used for description, and the Wilcoxon rank-sum test for complex sampling was used for analysis. In weighted logistic regression analysis, OR and their 95% confidence intervals (CI) were used to evaluate the relationship between DR and risk factors, with adjustment for confounding factors.

In the transcriptomic analysis, Strawberry Perl 5.32.1.1 was used to merge and annotate GEO datasets. For two independent samples, a t-test was applied; for two paired samples, the Wilcoxon signed-rank test was used; and for three or more groups, one-way analysis of variance (ANOVA) and Kruskal–Wallis rank-sum tests were applied. Spearman’s rank correlation test was used for correlation analysis. In the single-cell transcriptomic analysis, Seurat was used for normalization, dimensionality reduction, and clustering, with cell type annotation performed based on reference databases. Differential expression analysis focused on DEGs, and the Wilcoxon rank-sum test was applied to compare expression levels across cell types. A *p* value < 0.05 or a FDR < 0.05, corrected using the Benjamini–Hochberg method, was considered statistically significant.

All statistical analyses were performed using R 4.3.2.

## Result

### MR-based screening results for DR targets

**Fig. 2:**
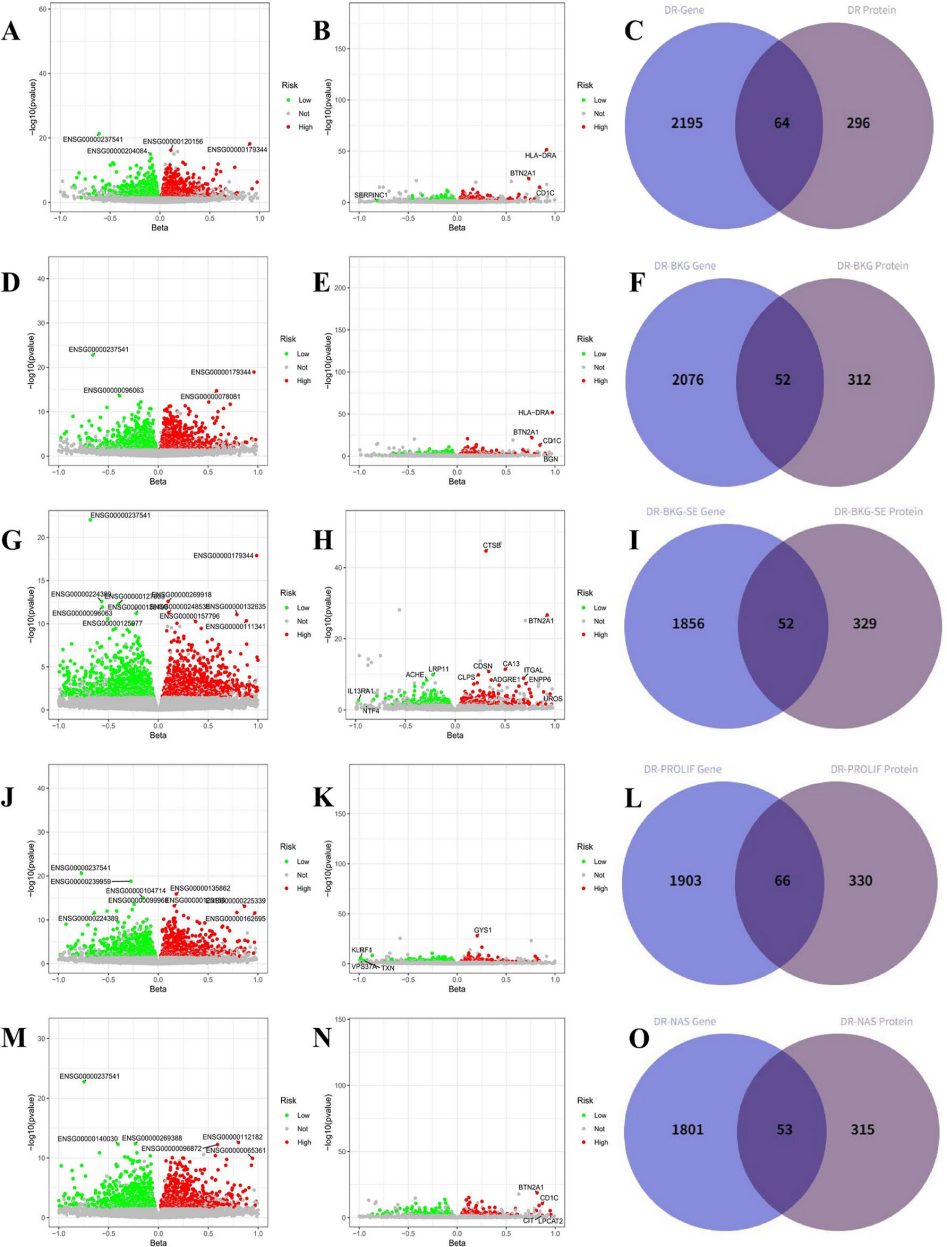
Volcano plots of MR results between eQtls and DR (**A**), DR-BKG (**D**), DR-BKG-SEVERE (**g**), DR-PROLIF (**J**), and DR-NAS (**M**); volcano plots of MR results between pQtls and DR (**B**), DR-BKG (**E**), DR-BKG-SEVERE (**H**), DR-PROLIF (**K**), and DR-NAS(**N**); shared gene-protein targets of DR (**C**), DR-BKG (**F**), DR-BKG-SEVERE (**I**), DR-PROLIF (**L**), and DR-NAS (**O**)

We performed MR analysis on the 15,711 genes selected and their association with various DR subtypes. After screening based on the adjusted FDR values and pleiotropy, the results showed that 2,553 genes (Fig. 2A and Supplementary File 1, Table S2) and 365 proteins (Fig. 2B and Supplementary File 1, Table S3) were causally associated with DR. Among these, 1,244 genes and 146 proteins were negatively associated with DR (protective factors), while 1,309 genes and 214 proteins were positively associated with DR (risk factors). Additionally, 64 targets (e.g., CA2, ABHD14B, ACADM) showed causal relationships with DR at both the gene and protein levels (Fig. 2C). For DR-BKG, 2,421 genes (Fig. 2D and Supplementary File 1, Table S4) and 364 proteins (Fig. 2Eand Supplementary File 1, Table S5) were causally associated, with 1,212 genes and 159 proteins negatively correlated, and 1,209 genes and 205 proteins positively correlated with DR-BKG. 52 targets (e.g., ACADM, ARSB, BST1) were identified with causal relationships at both the gene and protein levels for DR-BKG (Fig. 2F). For DR-BKG-SEVERE, 2,143 genes (Fig. 2G and Supplementary File 1, Table S6) and 381 proteins (Fig. 2H and Supplementary File 1, Table S7) were causally associated, with 1,073 genes and 180 proteins negatively correlated, and 1,070 genes and 201 proteins positively correlated with DR-BKG-SEVERE. 52 targets (e.g., C1QTNF6, CD8A, CLEC3B) showed causal relationships at both the gene and protein levels for DR-BKG-SEVERE (Fig. 2I). For DR-PROLIF, 2,230 genes (Fig. 2J and Supplementary File 1, Table S8) and 396 proteins (Fig. 2Kand Supplementary File 1, Table S9) were causally associated, with 1,104 genes and 206 proteins negatively correlated, and 1,126 genes and 190 proteins positively correlated with DR-PROLIF. 66 targets (e.g., BTN2A1, CD33, CDKN2D) showed causal relationships at both the gene and protein levels for DR-PROLIF (Fig. 2L). For DR-NAS, 2,089 genes (Fig. 2M and Supplementary File 1, Table S10) and 368 proteins (Fig. 2Nand Supplementary File 1, Table S11) were causally associated, with 1,042 genes and 174 proteins negatively correlated, and 1,047 genes and 194 proteins positively correlated with DR-NAS. 53 targets (e.g., CEP20, CLEC7A, COL15A1) showed causal relationships at both the gene and protein levels for DR-NAS (Fig. 2O).

### SMR analysis results of DR targets and DR

**Fig. 3:**
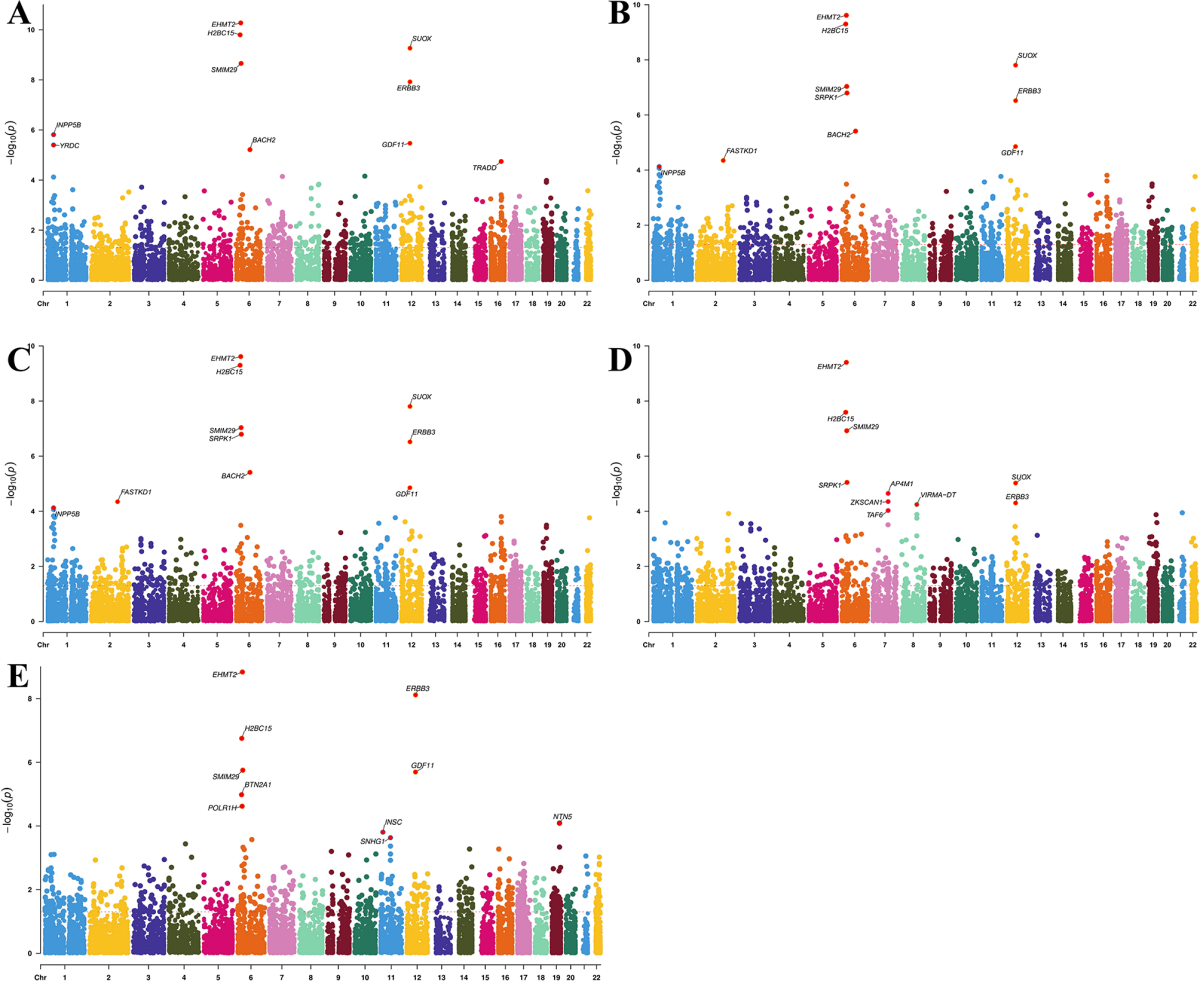
Manhattan plot of the SMR analysis between eQtls and DR (**A**), DR-BKG (**B**), DR-BKG-SEVERE (**C**), DR-PROLIF (**D**), and DR-NAS (**E**)

We used the SMR method to test 15,711 genes for their association with DR. The results showed that 981 genes were significantly associated with DR, with 462 genes and 25 proteins consistent with MR analysis results. Seventeen targets, including ACADM, PLCB2, and TP53INP1, overlapped between the gene and protein DR targets, as shown in Fig. 3A. Among 792 genes significantly associated with DR-BKG, 392 genes and 17 proteins were consistent with MR analysis results. Eight targets, including CEP170, ERBB3, and LAMP3, overlapped between the gene and protein DR-BKG targets, as shown in Fig. 3B. For DR-BKG-SEVERE, 766 genes were significantly associated, with 386 genes and 20 proteins consistent with MR analysis results. Thirteen targets, including C1QTNF6, CLEC3B, and CTSH, overlapped between the gene and protein DR-BKG-SEVERE targets, as shown in Fig. 3C. For DR-PROLIF, 895 genes were significantly associated, with 404 genes and 17 proteins consistent with MR analysis results. Twelve targets, including ACADM, ANKMY2, and CD209, overlapped between the gene and protein DR-PROLIF targets, as shown in Fig. 3D. For DR-NAS, 774 genes were significantly associated, with 366 genes and 17 proteins consistent with MR analysis results. Fourteen targets, including ANKMY2, APOBR, and BTN2A1, overlapped between the gene and protein DR-PROLIF targets, as shown in Fig. 3E. These results validated the robustness of some of our previous findings, and the HEIDI analysis showed no evidence of heterogeneity. Specific results are available in Supplementary File 1, Tables S12–S16. Given the multi-omics nature of this study involving gene-protein interaction networks and the multi-layered approach to risk factors and diseases, we prioritized selecting as many targets as possible for further analysis. SMR was used to assess the conditions for selecting the intermediary relationships of these targets.

### Analysis of risk factors and their association with DR based on NHANES cross-sectional data

#### Baseline characteristics

In the NHANES analysis, 946 participants were included, with a weighted median age of 61 years. Because DR status in NHANES is ascertained only among respondents with diagnosed DM, our analytic cohort comprises adults with DM; therefore, group comparisons reflect DM with DR vs DM without DR. Among them, 49% were male and 51% were female. There were 194 DR participants (weighted percentage of 18%). Table 1 presents the group comparison between DR and non-DR. Among continuous variables, age showed a statistically significant difference between groups (*p* = 0.018), with DR patients being older. There was a significant statistical difference in poverty level between groups (*p* < 0.001), with DR being more common among those in poverty. There were significant differences in the presence of HBP, Stroke, and renal insufficiency between the DR and non-DR groups (*p* values of 0.004, 0.009, and < 0.001, respectively), with a higher proportion of DR patients among those with HBP, Stroke, and renal insufficiency. Cr and BUN levels were significantly higher in the DR group compared to the non-DR group (*p* values of 0.01 and < 0.001, respectively), while DBP was significantly lower in the DR group compared to the non-DR group.

**Table 1:** Baseline characteristics of the NHANES population

		Diabetic retinopathy			
Characteristic	**N**^1^	**Overall**, *N* = 946 (100%)^2^	**No**, *N* = 752 (82%)^2^	**Yes**, *N* = 194 (18%)^2^	*p* **Value**^3^
**Gender**	946				> 0.9
male		476 (49%)	376 (49%)	100 (50%)	
female		470 (51%)	376 (51%)	94 (50%)	
**Age**	946	61 (50, 71)	61 (50, 70)	64 (52, 73)	**0.018**
**Age (year) Group**	946				0.066
65+ years		497 (44.38%)	388 (42.20%)	109 (54.36%)	
40–64 years		396 (47.02%)	322 (48.91%)	74 (38.35%)	
15–39 years		53 (8.60%)	42 (8.89%)	11 (7.28%)	
**Race**	946				0.8
Non-Hispanic White		391 (66%)	315 (67%)	76 (63%)	
Non-Hispanic Black		222 (14%)	178 (14%)	44 (15%)	
Mexican American		222 (8.6%)	179 (8.5%)	43 (9.1%)	
Other Race		31 (5.8%)	21 (5.5%)	10 (7.1%)	
Other Hispanic		80 (4.9%)	59 (4.7%)	21 (5.8%)	
**Education**	946				0.10
Less Than 9th Grade		218 (13%)	166 (12%)	52 (19%)	
9-11th Grade		185 (16%)	146 (15%)	39 (17%)	
High School Grad/GED		214 (27%)	171 (27%)	43 (29%)	
Some College or AA degree		215 (28%)	169 (29%)	46 (27%)	
College Graduate or above		114 (15%)	100 (17%)	14 (8.8%)	
**Poverty**	946				** < 0.001**
Not poor		609 (76%)	502 (80%)	107 (59%)	
poor		337 (24%)	250 (20%)	87 (41%)	
**Alcohol**	946				0.10
Non-drinker		377 (38%)	295 (36%)	82 (45%)	
Alcohol consumer		569 (62%)	457 (64%)	112 (55%)	
**Smoke**	946				> 0.9
Never smoker		479 (51%)	381 (51%)	98 (50%)	
Smoker		467 (49%)	371 (49%)	96 (50%)	
**BMI**	946	31 (27, 37)	31 (27, 37)	30 (27, 36)	0.6
**BMI(kg/m^2) Group**	946				0.4
Normal (18.5 to < 25)		119 (14%)	97 (14%)	22 (12%)	
Underweight ( < 18.5)		3 (0.2%)	3 (0.3%)	0 (0%)	
Overweight (25 to < 30)		295 (27%)	232 (25%)	63 (32%)	
Obese (30 or greater)		529 (60%)	420 (60%)	109 (56%)	
**Hypertension**	946				**0.004**
non-HBP		234 (28%)	196 (30%)	38 (17%)	
HBP		712 (72%)	556 (70%)	156 (83%)	
**Diabetes Duration (Month)**	946	504 (12, 672)	504 (12, 672)	480 (12, 624)	0.5
**Glycemic Control**	946	550 (55%)	420 (54%)	130 (62%)	0.2
**STROKE**	946	104 (9.9%)	69 (8.4%)	35 (17%)	**0.009**
**CHD**	946	147 (15%)	109 (15%)	38 (17%)	0.6
**Weak Kidney**	946	76 (6.3%)	41 (4.3%)	35 (15%)	** < 0.001**
**HbA1c**	946	6.80 (6.10, 7.70)	6.80 (6.10, 7.60)	7.00 (6.10, 7.90)	0.089
**GLU**	946	134.00 (112.50, 172.00)	133.00 (112.80, 172.00)	142.10 (111.00, 179.40)	0.5
**HDL-c**	946	1.22 (1.03, 1.50)	1.22 (1.03, 1.50)	1.20 (1.06, 1.47)	0.7
**LDL-c**	946	2.56 (2.04, 3.31)	2.59 (2.07, 3.31)	2.51 (1.97, 3.26)	0.4
**TG**	946	1.56 (1.11, 2.24)	1.52 (1.10, 2.18)	1.76 (1.17, 2.35)	0.3
**TC**	946	4.65 (4.03, 5.48)	4.71 (4.06, 5.46)	4.50 (3.98, 5.53)	0.5
**Cr**	946	75.14 (61.88, 90.17)	73.37 (61.88, 88.40)	79.56 (64.53, 111.38)	**0.010**
**BUN**	946	5.00 (3.93, 6.78)	5.00 (3.93, 6.43)	6.07 (4.28, 9.28)	** < 0.001**
**CRP**	946	0.27 (0.11, 0.62)	0.28 (0.11, 0.64)	0.22 (0.14, 0.53)	0.9
**SBP**	946	127.33 (116.00, 139.33)	127.33 (116.00, 139.00)	128.67 (114.00, 144.00)	0.6
**DBP**	946	69.33 (59.33, 76.67)	70.00 (60.00, 76.67)	67.33 (58.00, 73.33)	**0.022**

N not Missing (unweighted)

median (IQR) for continuous; n (%) for categorical

Pearson’s X^2: Rao & Scott adjustment; Design-based Kruskal-Wallis test

Note: The analytic sample includes NHANES participants with diagnosed diabetes only. “Yes” denotes DR among individuals with diabetes; “No” denotes diabetes without DR. All analyses apply complex survey weights, strata, and PSUs

#### Analysis of the association between risk factors and DR based on logistic regression and restricted cubic spline (RCS)

**Fig. 4:**
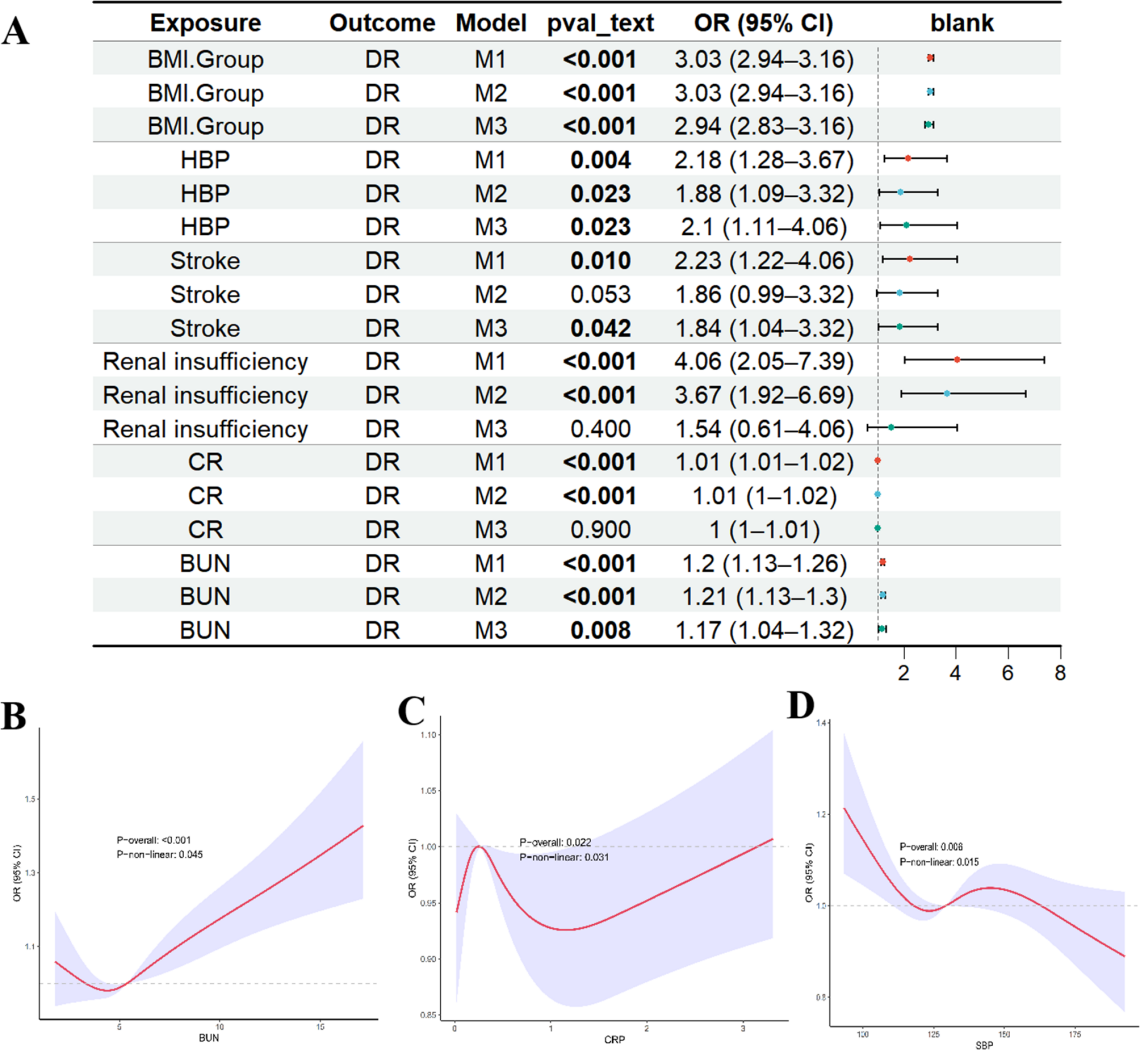
(**A**) Forest plot of significant DR risk factors based on NHANES; (**B**) RCS curve of BUN; (**C**) RCS curve of CRP; (**D**) RCS curve of SBP

The NHANES analysis performed a complex sampling-weighted logistic regression analysis for DR as the outcome and other variables as exposures. The analysis was based on three models: a univariate logistic analysis for exposure and outcome, a multivariate logistic model including demographic variables (gender, age, race), and a multivariate logistic model including all variables. Additionally, continuous variables were analyzed using RCS to assess their nonlinear relationships, with the results presented in a forest plot as Fig. 4A.

In the three models with BMI as the exposure, compared to the normal group, the risk of DR in the overweight group was significantly increased by 2.94–3.03 times (*p* < 0.001). For HBP as the exposure, all three models showed a significant association with the outcome (*p* values were 0.004, 0.023, and 0.023, respectively). In Models 1, 2, and 3, the risk of DR in the HBP group was 2.18 times, 1.88 times, and 2.1 times higher, respectively, compared to those without HBP. For Stroke as the exposure, Model 1 and Model 3 indicated a significant association with the outcome (*p* values of 0.01 and 0.042, respectively), with a 2.23 times and 1.84 times higher risk of DR in the Stroke group compared to non-Stroke patients. For renal insufficiency as the exposure, Model 1 and Model 2 showed a significant association with the outcome (*p* < 0.001), with the risk of DR in the renal insufficiency group being 4.06 times and 3.67 times higher compared to those without renal insufficiency. For Cr as the exposure, Models 1 and 2 (*p* < 0.001) indicated that for every 1 μmol/L increase in Cr, the risk increased by 1%. For BUN as the exposure, all three models showed statistical significance (*p* values of < 0.01, < 0.01, and 0.008, respectively), with the risk of DR increasing by 17%-21% for every 1 mmol/L increase in BUN. Based on the RCS analysis in Model 3 (Fig. 4B), there was a significant non-linear relationship between BUN and DR (*p*-non-linear = 0.045). Within the range of 2.5–5 mmol/L for BUN, it was inversely related to DR risk, while outside this range, the relationship was positively correlated with DR risk. Additionally, RCS analysis in Model 3 for CRP (Fig. 4C) indicated a significant non-linear relationship between CRP and DR, with CRP below 3 mg/dL showing a negative association with DR. RCS analysis for SBP as the exposure in Model 3 (Fig. 4D) indicated a significant non-linear relationship between SBP and DR, with a positive correlation between SBP and DR in the range of 130,160 mmHg. No significant associations were found for other variables.

### Two-sample MR analysis of risk factors and DR subtypes and results of risk factor target screening

**Fig. 5:**
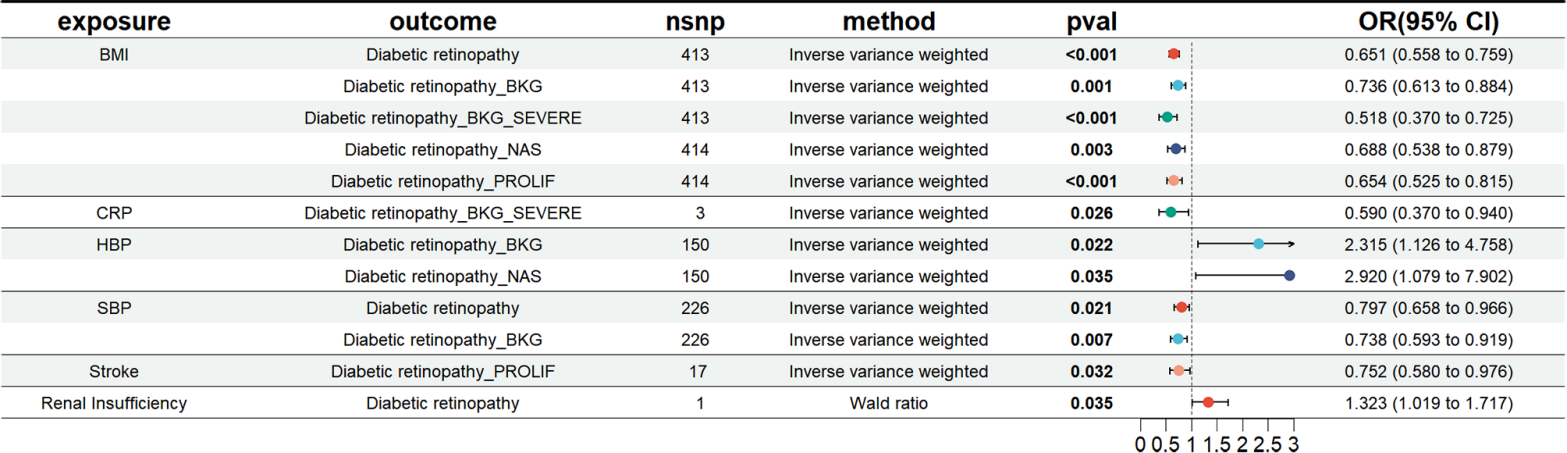
Forest plot of significant DR risk factors based on MR

We conducted a MR analysis with all the risk factors involved in NHANES and five DR subtypes as outcomes to validate and extend the observational analysis results. The MR results between risk factors and DR subtypes showed significant causal relationships for BMI, CRP, HBP, SBP, and Stroke with DR subtypes, as shown in Fig. 5. These five risk factors were identified in the NHANES analysis, and the directions for HBP, CRP, and SBP were consistent with NHANES results, whereas the direction for Stroke was opposite, showing a significant negative association. At the subtype level, BMI had a negative causal relationship with all five DR subtypes, CRP had a negative causal relationship with DR-BKG, HBP showed a positive causal relationship with both DR-BKG and DR-NAS, SBP had a negative causal relationship with both DR and DR-BKG, and Stroke had a negative causal relationship with DR-PROLIF.

### MR analysis results of eQTL and pQTL with significant risk factors

**Fig. 6:**
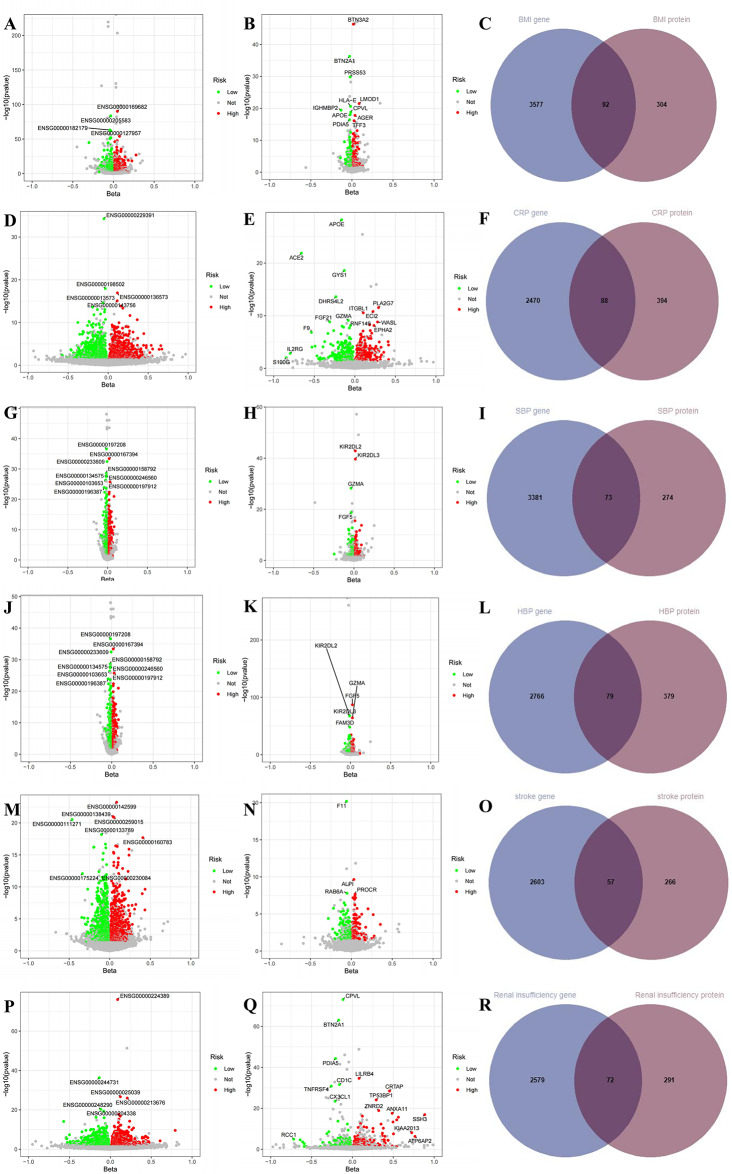
Volcano plots of MR results between eQtls and BMI (**A**), CRP (**D**), SBP (**G**), HBP (**J**), Stroke (**M**), and renal insufficiency (**P**); Volcano plots of MR results between pQtls and BMI (**B**), CRP (**E**), SBP (**H**), HBP (**K**), Stroke (**N**), and renal insufficiency (**Q**); Shared gene-protein targets of BMI (**C**), CRP (**F**), SBP (**I**), HBP (**l**), Stroke (**O**), and renal insufficiency (**R**)

We further conducted target screening for the significant risk factors with a causal association to DR subtypes as described in section “MR-Based screening results for dr targets”, identifying targets that regulate the positive risk factors for DR. Specifically, we found 4,237 genes related to BMI (Fig. 6A), 396 related proteins (Fig. 6B), and 92 gene-protein overlapping targets such as AAMDC, ABO, and ACP6 (Fig. 6C). For CRP, we identified 2,913 genes (Fig. 6D), 482 related proteins (Fig. 6E), and 88 overlapping gene-protein targets including ADAM22, ADAMTSL4, and AKR1B1 (Fig. 6F). For SBP, 3,960 genes (Fig. 6G) and 347 related proteins (Fig. 6H) were identified, with 73 overlapping targets at the gene-protein level such as ACP1, ACP6, and AFAP1 (Fig. 6I). For HBP, we found 3,221 genes (Fig. 6J) and 458 proteins (Fig. 6K), with 79 overlapping targets such as AAMDC, ACP6, and ADAM23 (Fig. 6L). For Stroke, there were 3,221 genes (Fig. 6M) and 458 proteins (Fig. 6N), with 57 overlapping targets at the gene-protein level including ABO, AOC1, and ARL2BP (Fig. 6O). Finally, for Renal insufficiency, 3,041 genes (Fig. 6P) and 363 related proteins (Fig. 6Q) were identified, with 72 gene-protein overlapping targets such as ADAM15, CD86, and AARSD1 (Fig. 6R). The MR results for all risk factors and outcomes are presented in Supplementary File 1, Table S17.

### Transcriptome expression analysis results of common targets between risk factors and DR

**Fig. 7:**
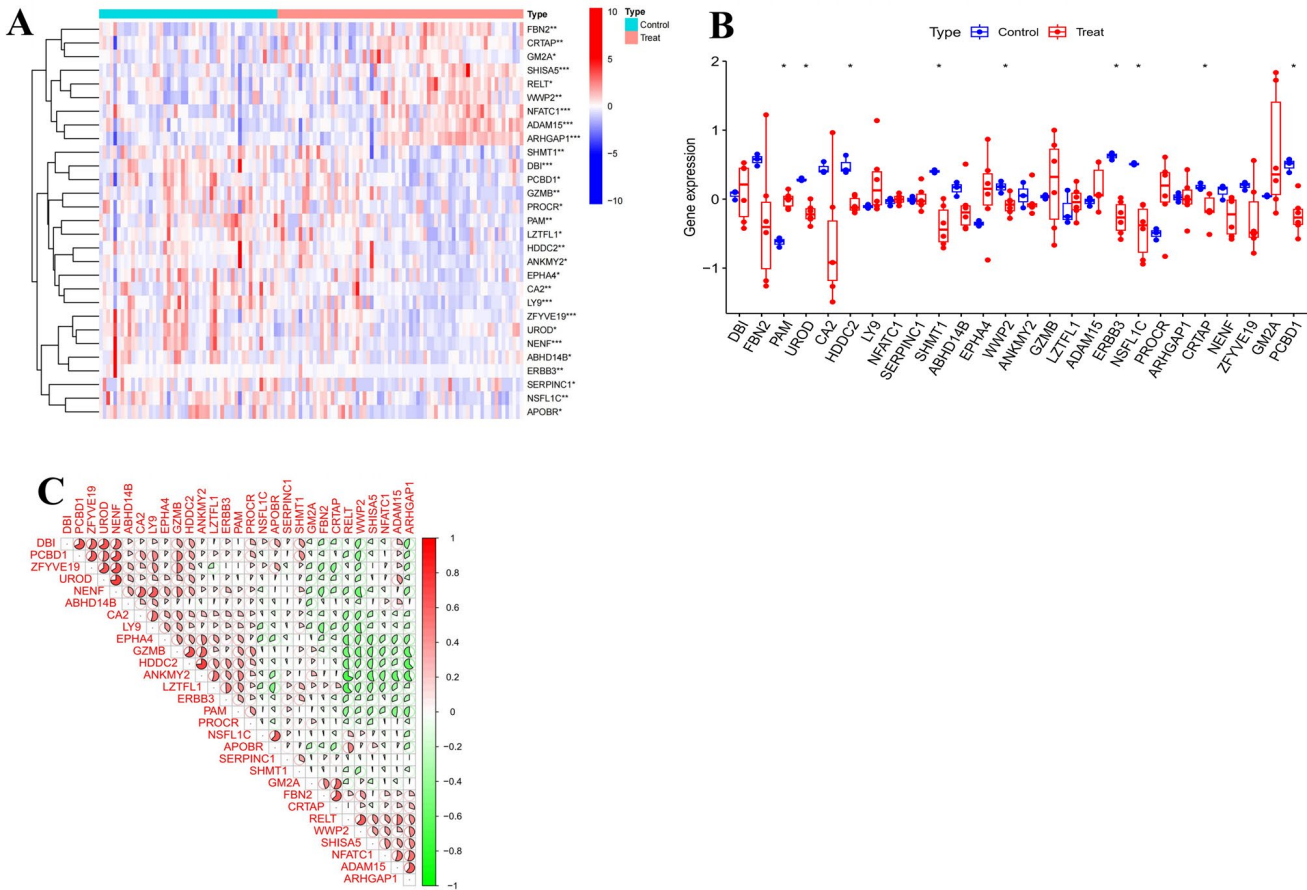
(**A**) Heatmap of expression difference analysis of common targets between risk factors and DR genes between control group and DR group; (**B**) Validation of expression differences in common targets of risk factor–associated genes and DR-related genes between control and DR groups (box plot); (**C**) SDETGs correlation network

We intersected the targets related to the six significant risk factors with the targets related to DR subtypes, resulting in 403 targets that can simultaneously regulate both the risk factors and DR subtypes for transcriptome expression analysis. Differential analysis of peripheral blood samples between the DR group and the normal group revealed 29 significant differentially expressed treatment targets (SDETGs) for DR. Among these genes, FBN2, CRTAP, GM2A, SHISA5, RELT, WWP2, NFATC1, ADAM15, and ARHGAP1 were highly expressed in the DR group, while SHMT1, DBI, PCBD1, GZMB, PROCR, PAM, LZTFL1, HDDC2, ANKMY2, EPHA4, CA2, LY9, ZFYVE19, UROD, NENF, ABHD14B, ERBB3, SERPINC1, NSFL1C, and APOBR were highly expressed in the normal group, as shown in Fig. 7A. To further validate these findings, we performed transcriptome differential expression analysis of the SDETGs in retinal samples. The analysis identified nine genes with statistically significant differences between the DR and control groups. Among them, NFATC1, ADAM15, and GM2A were highly expressed in the DR group, consistent with the results observed in peripheral blood. In contrast, DBI, PAM, LY9, EPHA4, GZMB, LZTFL1, and PROCR were also highly expressed in the DR group, while the remaining genes were more highly expressed in the control group, as shown in Fig. 7B. Correlation analysis between each pair of SDETGs in the DR samples indicated strong correlations between SDETGs, with both positive and negative correlations being significant, as shown in Fig. 7C. Detailed information on the shared gene expression between risk factors and DR is provided in Supplementary File 1, Table S18.

### ScRNA-seq analysis results

**Fig. 8:**
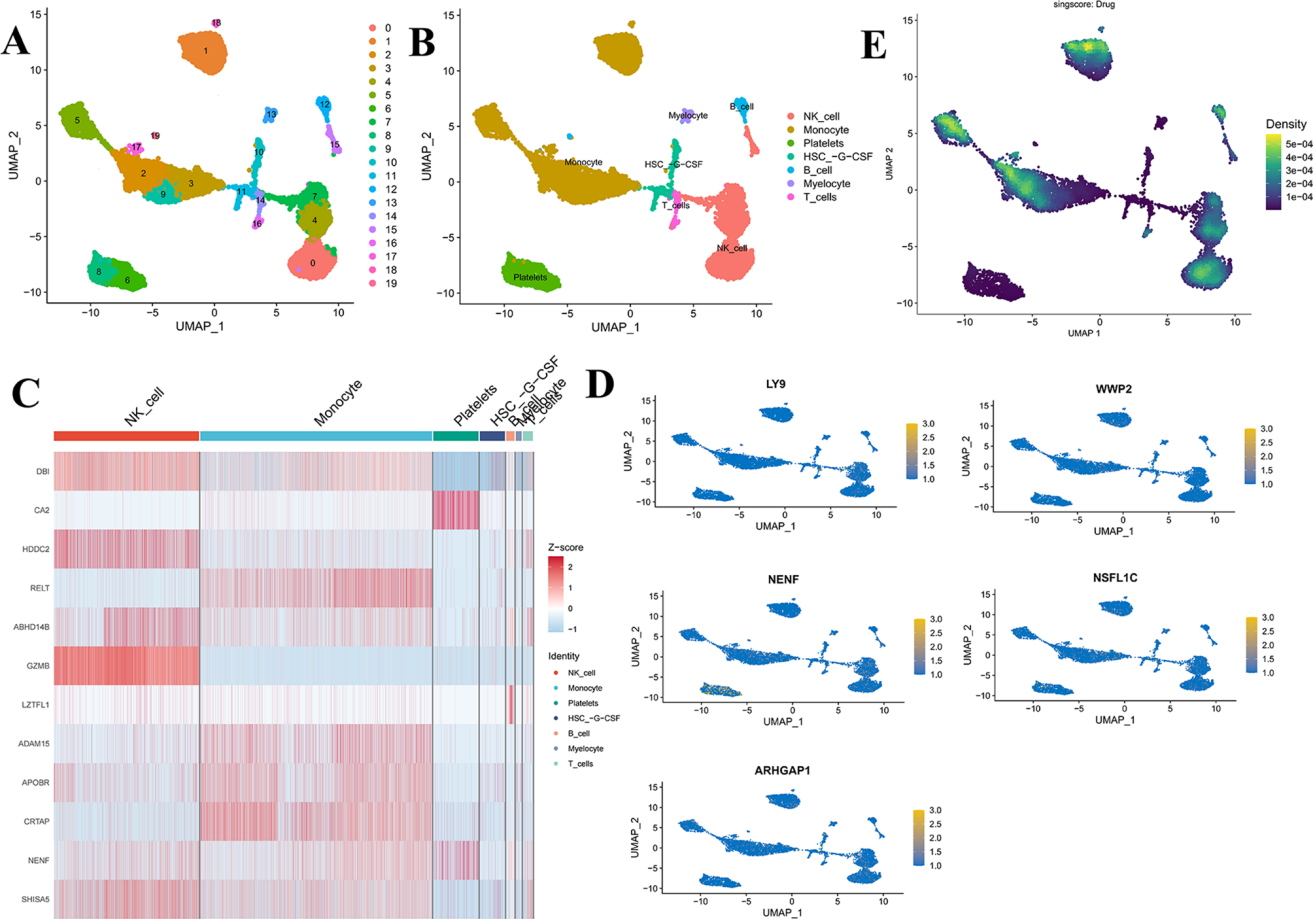
(**A**) Uniform manifold approximation and projection (UMAP) visualization of 20 clusters from scRNA-seq; (**B**) UMAP showing annotated cell types; (**C**) Heatmap showing the expression patterns of 29 SDETGs across annotated cell types; (**D**) UMAP feature plots of five feature targets (LY9, WWP2, nenf, NSFL1C, ARHGAP1) with distinct distributions in NK cells, monocytes, and platelets; (**E**) UMAP visualization of drug pathway activity scores (singscore: drug), highlighting enriched activity in monocytes and T cells

ScRNA-seq analysis identified 20 clusters, which were annotated into major immune and hematopoietic populations (Fig. 8A–B). Among the 29 SDETGs, several genes exhibited particularly prominent expression patterns at the single-cell level, including DBI, CA2, HDDC2, RELT, ABHD14B, GZMB, LZTFL1, ADAM15, APOBR, CRTAP, NENF, and SHISA5 (Fig. 8C). For example, ADAM15 was enriched in monocytes, GZMB in natural killer (NK) cells, DBI and CA2 in NK cells and platelets, and NENF in platelets. In parallel, we highlighted the five feature targets derived from the machine learning model (LY9, WWP2, NENF, NSFL1C, ARHGAP1), which showed distinct cellular distributions: LY9 in NK cells, WWP2 in monocytes, NENF in platelets, NSFL1C in monocytes and NK cells, and ARHGAP1 in monocytes and NK cells (Fig. 8D). Drug pathway scoring analysis (singscore) further revealed that drug-associated gene sets displayed higher activity in monocytes and T cells, suggesting potential cell-type-specific drug responsiveness (Fig. 8E). Detailed single-cell expression values of the 29 SDETGs across different cell types are provided in Supplementary File 1, Table S19.

### Immune cell analysis results in DR samples

**Fig. 9:**
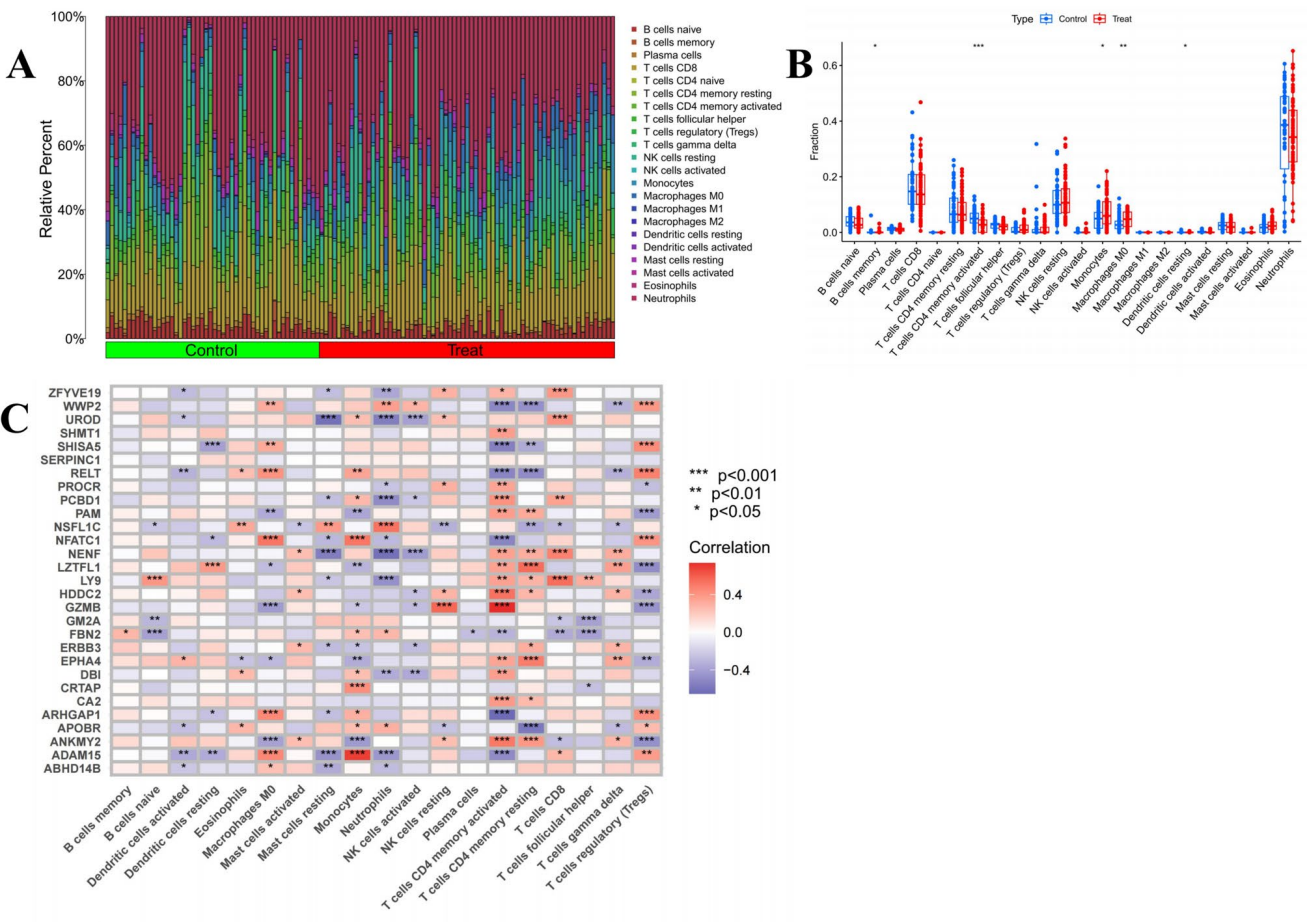
(**A**) Bar plot of relative percentage of each immune cells in samples; (**B**) Box plot of immune cell fraction between control group and DR group; (**C**) Heatmap of correlation analysis between SDETGs and immune cells

Immune cell infiltration analysis was performed to determine the types and amounts of immune cells expressed in each sample (Fig. 9A). ssGSEA was then conducted to identify immune cells with statistically significant expression in the DR group and the control group. Memory B cells, activated CD4+ memory T cells, and resting dendritic cells were significantly more highly expressed in the control group, while monocytes and M0 macrophages were highly expressed in the DR group, as shown in Fig. 9B. Correlation analysis between immune cells and SDETG of DR (Fig. 9C) revealed a strong relationship, with both positive and negative regulatory associations being significant. Immune cell expression profiles for each sample are provided in Supplementary File 1, Table S20.

### SDETG enrichment analysis results

**Fig. 10:**
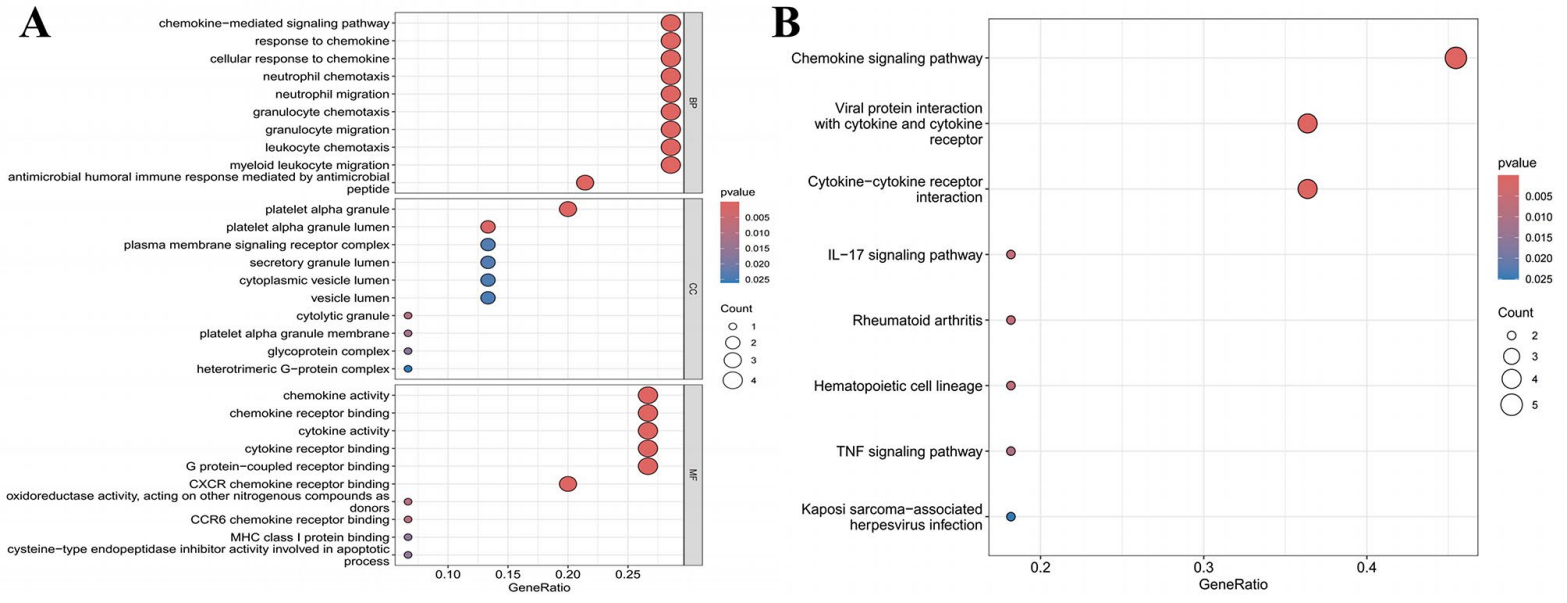
Bubble plot of enrichment analysis. (**A**) Go enrichment analysis; (**B**) KEGG pathway enrichment analysis

Enrichment analysis of the biological functions and pathways of DRs SDETG was conducted to further enrich the mechanistic findings. The GO analysis revealed that the main biological processes involved the regulation of chemokines and immune cells, including the chemokine-mediated signaling pathway, cellular response to chemokines, neutrophil chemotaxis, leukocyte chemotaxis, etc., as shown in Fig. 10A. The KEGG pathway enrichment analysis similarly indicated the involvement of chemokine, cytokine, and immune-inflammatory related pathways, including the Chemokine signaling pathway, TNF signaling pathway, IL-17 signaling pathway, and others, as shown in Fig. 10B. The specific results of the GO and KEGG enrichment analyses are presented in Supplementary File 1, Table S21.

### Machine learning model and nomogram selection results for DR’s SDETGs

**Fig. 11:**
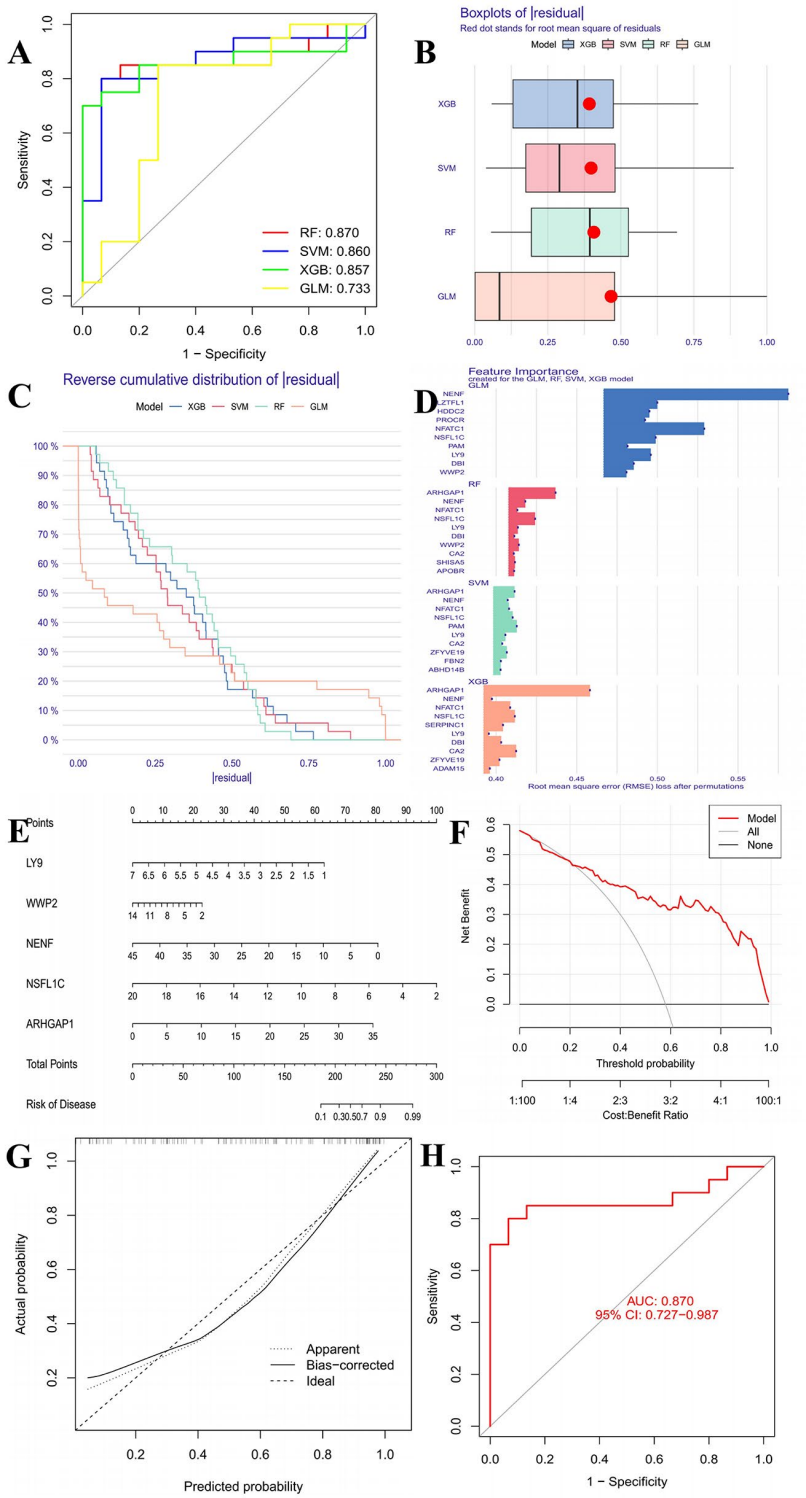
(**A**) ROC of the four machine learning models; (**B**) Box plots of residual of the four machine learning models; (**C**) Reverse cumulative distribution of residual; (**D**) Bar plot of feature importance of the four machine learning models; (**E**) Nomogram of the feature genes; (**F**) Decision curve of feature genes nomogram; (**G**) Calibration curve of feature genes nomogram; (**H**) ROC of the validation geo dataset

We constructed a machine learning prediction model using the SDETG data for DR. By analyzing the residual box plots, reverse cumulative distribution plots, and ROC curves, we found that the RF model for the DR group exhibited higher accuracy. The model’s ROC curve had a larger area under the curve (AUC) (Fig. 11A), and the residual and reverse cumulative values were lower (Figs. 11B–11C). Therefore, we selected RF as the optimal model. We obtained the importance scores of the feature genes using this model (Fig. 11D). Using the top five feature targets (LY9, WWP2, NENF, NSFL1C, ARHGAP1), we built a nomogram (Fig. 11E). By calculating the total expression score of the feature targets, we predicted the risk rate of DR feature targets in DR development, which helped determine treatment sensitivity. From the calibration curve (Fig. 11F) and decision curve (Fig. 11G), it is evident that the prediction accuracy is high. In retinal samples as supplementary validation, the RF model achieved an AUC of 0.870, indicating that the model maintained high robustness and accuracy (Fig. 11H).

### Two-step mediation MR analysis results of target-risk factor-DR

**Fig. 12:**
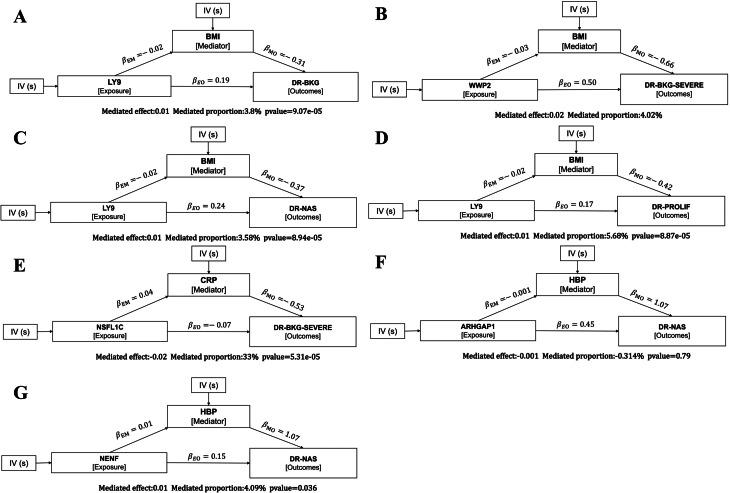
Results of mediation Mendelian randomization analysis. (**A**) LY9→BMI→DR-BKG; (**B**) WWP2→BMI→DR-BKG-SEVERE; (**C**) LY9→BMI→DR-NAS; (**D**) LY9→BMI→DR-PROLIF; (**E**) NSFL1C→CRP→DR-BKG-SEVERE; (**F**) ARHGAP1→HBP→DR-NAS; (**G**) NENF→HBP→DR-NAS

To further investigate the indirect effects of target genes on DR outcomes via risk factors, we conducted mediation analysis using effect estimates from two-step MR and the total effects from primary MR analysis. The exposures included five characteristic targets (LY9, WWP2, NENF, NSFL1C, ARHGAP1) that showed significant associations with both risk factors and various DR subtypes. Indirect effects were estimated using the product method, and standard errors (SEs) and CIs were calculated using the delta method. LY9 was negatively associated with BMI, which in turn was negatively associated with DR-BKG, DR-NAS, and DR-PROLIF, resulting in an overall positive mediating effect of LY9 on these DR subtypes. The proportion of the mediation effect to the total effect was 3.8%, 3.58%, and 5.68%, respectively (Figs. 12A, 12C, and 12D). WWP2 influenced DR-BKG-SEVERE via BMI, with a mediation proportion of 4.02% (Fig. 12B). NSFL1C positively regulated CRP, which was negatively associated with DR-BKG-SEVERE, resulting in a total negative regulatory effect, with a mediation proportion of 33% (Fig. 12E). In addition, both ARHGAP1 and NENF were positively associated with DR-NAS through HBP as the mediator (Figs. 12F and 12G).

## Discussion

In this study, we systematically integrated large-scale multi-omics data, MR, transcriptomic analysis, immune infiltration analysis, and machine learning techniques to identify potential pathogenic genes, regulatory pathways, and key intermediary mechanisms associated with DR and its subtypes. We aimed to uncover the underlying etiological network and regulatory patterns involved in the development of DR, providing theoretical insights and research directions for the personalized diagnosis and treatment of DR.

### Associated factors: epidemiologic and genetic evidence

We evaluated DR risk factors from two complementary perspectives: a NHANES cross-sectional analysis restricted to individuals with diagnosed DM (comparing DR vs. non-DR within DM) and two-sample MR capturing lifelong genetic predisposition. The results indicate that BMI, HBP, SBP, CRP, stroke, and renal insufficiency are associated with DR.

Prior work has established that higher BMI promotes the onset and progression of DM [15]. In our study, NHANES showed a positive association between BMI and DR within the DM population, whereas MR yielded inverse directions for certain DR subtypes. The literature on the BMI–DR relationship is likewise inconsistent: a meta-analysis of 59 cohorts reported no significant association [16]; other cohorts observed lower DR risk at higher BMI, in line with our MR direction [17]; while still other studies support BMI as a risk factor, consistent with our NHANES findings. These discrepancies likely reflect two main issues: (i) differences in study populations and design—cross-sectional/cohort analyses typically compare within DM and are susceptible to selection and treatment effects, whereas MR reflects the lifelong genetic effect of adiposity; and (ii) construct validity—BMI does not distinguish visceral adiposity, fat distribution, or sarcopenic obesity, and subgroup evidence suggests that sarcopenic obesity, rather than generalized obesity, may be the phenotype that increases DR risk [18].

Large randomized trials and long-term cohort studies [19–21] consistently show that sustained glycemic control markedly lowers the incidence and progression of DR, indicating that chronic hyperglycemia is a key driver. In our NHANES analyses (GLU, HbA1c, antidiabetic medication use, diabetes duration) and in two-sample MR, we did not observe stable associations. Likely explanations include: (i) cross-sectional data capture only a single measurement and thus poorly reflect long-term glycemic exposure; (ii) patients with established DR are more likely to receive intensive therapy, which can lower current HbA1c and bias associations toward the null; and (iii) selection biases intrinsic to questionnaire-based data. Therefore, we do not dispute the causal role of long-term glycemic control in DR.

HBP is a known DR risk factor, and research suggests that hypertension, especially severe DR, is associated with salt-sensitive corticosteroid receptor overactivation [22]. SBP is considered a more important factor for retinal damage than hyperglycemia [23]. CRP is often considered positively correlated with DR onset in previous studies, and animal experiments have shown that increased CRP levels contribute to the pathogenesis of DR. This effect is associated with the overexpression of pro-inflammatory, pro-oxidative, and pro-angiogenic factors, as well as upregulation of CD32 and NF-κB signaling in the retina [24, 25]. Our NHANES and MR results contradict these findings, with one study suggesting that when CRP levels exceed 10.11 mg/L, the likelihood of DR increases, which may explain the results observed in a specific range [26]. Stroke is correlated with DR risk, and stroke is a contributing factor to DR, while DR is a predictor of stroke outcomes [27, 28]. Renal dysfunction and renal-related indicators (such as Cr, BUN) are associated with DR prevalence, increasing both the incidence and progression of DR [26].

Although nutrients and vitamins were not explicitly modeled as exposures in this study, they likely play important roles in the development and progression of DR and sit at key nodes of the immunometabolic pathways implicated in its pathogenesis. Recent Mendelian randomization work suggests potential causal links between several vitamins and diabetic complications, providing leads for nutritional intervention strategies [29]. In the domain of lipid nutrition, a prospective analysis from PREDIMED reported that higher intake of marine long-chain *n*-3 polyunsaturated fatty acids was associated with a lower incidence of vision-threatening DR among individuals with type 2 diabetes, pointing to protective anti-inflammatory/pro-resolving mechanisms [30]. In parallel, systematic reviews and meta-analyses indicate that overall dietary patterns show favorable associations with DR risk, supporting a food-based, comprehensive approach to prevention [31]. Given that our work primarily focused on metabolic and inflammatory pathways, future research—within prospective cohorts and genetic causal frameworks—should further evaluate the effects of vitamins and nutrients across DR subtypes and immunometabolic pathways to assess their translational potential.

### Therapeutic targets: multi-omics findings and translational potential

Based on MR and transcriptomic analysis, we identified targets with differential expression that are common to risk factors and DR, which are derived from multi-source data comparisons and have relatively high clinical significance. Among these, FBN2, which encodes fibrillin 2, is a component of microfibrils that supports the eye lens, nerves, muscles, and more, containing transforming growth factor proteins that affect the growth and repair of systemic tissues [32]. Animal experiments have shown that this protein is involved in the fibrotic process of DR [33]. NFATC1 encodes nuclear factor of activated T-cells 1, which participates in the calcium/calmodulin-dependent protein kinase-NFAT signaling cascade. Studies have shown that inhibiting the expression of this target can reduce mitochondrial fission and cell apoptosis, thereby exerting a therapeutic effect on DR [34]. ADAM15 encodes ADAM metallopeptidase domain 15, which is involved in various processes of cell adhesion. In a mouse model of DR, this target increased endothelial permeability and enhanced the response to thrombin, promoting pathological neovascularization in the retina [35]. DBI encodes diazepam-binding inhibitor, and studies have shown that the known DR therapeutic drug anti-VEGF works by modulating the DBI-related pathways, thereby exerting beneficial effects on retinal glial cell inflammation and neurotrophy [36]. PROCR encodes protein C receptor, and studies have confirmed that the protein’s association with heme metabolism is related to pathological angiogenesis, making it a potential target for proliferative DR [37]. PAM encodes peptide glycine α-amidating monooxygenase, which is associated with type 2 diabetes risk in European populations, and similar studies have indicated that PAM is a potential treatment target for DR [38]. CA2 encodes carbonic anhydrase 2, and its inhibitors can reduce intraocular pressure, making it a potential treatment for DR [39]. ERBB3 encodes ERB-b2 receptor tyrosine kinase 3, and gene polymorphism and multi-omics studies have found that ERBB3 is related to diabetes and is a potential DR therapeutic target [40]. APOBR encodes apolipoprotein B receptor, and retrospective clinical studies have found that ApoB levels are higher in the DR group [41], while proteomics has identified ApoB as a biomarker for DR with potential to elucidate the molecular mechanisms of DR [42].

Other differential targets have not been studied in relation to DR, making them new findings in this research. Particularly, the high-importance DR feature targets identified by machine learning, including LY9, WWP2, NENF, NSFL1C, and ARHGAP1, warrant further study. LY9 (SLAMF3) is an immune coreceptor regulating T/NK-cell activation and cytokine release, thereby shaping the inflammatory milieu that can interface with adiposity and downstream microvascular injury (consistent with its methylation signal in diabetic nephropathy) [43, 44]. WWP2 is an E3 ubiquitin ligase; experimental data show that reduced WWP2 aggravates diabetic endothelial injury by promoting DDX3X ubiquitination and degradation, linking obesity-related metabolic stress to endothelial barrier dysfunction [45, 46]. ARHGAP1, a GAP for Rho GTPases, connects to the RhoA-ROCK axis that controls endothelial contractility, permeability, leukostasis and vascular tone, offering a plausible conduit to blood-pressure-related risk [47, 48]. NSFL1C (p47), a co-factor of the AAA+ ATPase p97/VCP, participates in proteostasis and intersects with NF-κB-mediated inflammatory stress, providing a mechanistic rationale for its alignment with systemic inflammatory markers such as CRP [49]. NENF (neudesin) modulates energy balance and adipose/neuronal-humoral regulation and may influence microvascular vulnerability through hemodynamic and immunometabolic pathways [47]. Consistent with these biological roles, our two-step mediation MR suggested LY9→BMI→DR-BKG/DR-NAS/DR-PROLIF, WWP2→BMI→DR-BKG-SEVERE, NSFL1C→CRP→DR-BKG-SEVERE, and ARHGAP1/NENF→HBP→DR-NAS as indirect causal chains. Taken together with our pathway and immune-infiltration analyses, which highlighted chemokine/cytokine and immune-inflammatory programs [50], these data position LY9/NSFL1C at immune–inflammation hubs, WWP2/ARHGAP1 at endothelial–cytoskeletal/vascular-dynamics nodes, and NENF at energy-metabolic control points, collectively supporting target→risk-factor→DR-subtype mechanistic pathways.

### Relationships among DR, DM, and other diabetic complications

DR is not only a prototypical microvascular complication of DM but also closely interconnected—temporally and pathophysiologically—with other diabetic complications via a shared “common soil,” including chronic hyperglycemia, elevated blood pressure, immuno-inflammatory activation, and metabolic dysregulation. Epidemiologic evidence indicates cross-organ predictability: for example, even short-term duration of DR predicts subsequent diabetic kidney disease, suggesting continuity or synchrony of microvascular injury across organs [51]. Consistent with this shared-soil concept, the Steno-2 randomized trial demonstrated that intensive multifactorial intervention (targets for glycemia, blood pressure, lipids, and lifestyle) substantially reduced micro- and macrovascular complications and all-cause mortality, with durable benefits on long-term follow-up [52, 53]. Likewise, in the FIELD Eye substudy, fenofibrate reduced the need for laser treatment and slowed retinopathy progression—supporting that systemic lipid-pathway modulation can favorably influence retinal outcomes [54]. Beyond classical cardiometabolic drivers, nutrients and vitamins likely sit at key nodes of the immunometabolic network implicated in complications. Recent MR suggests potential causal links between several vitamins and diabetic complications, providing leads for targeted nutritional strategies [29]. At a systems level, causal inference also points to bidirectional ties between DM and cancer risk (e.g., pancreatic cancer), highlighting how a pro-inflammatory, dysmetabolic milieu may extend beyond microvascular disease to broader organ susceptibility [55]. In line with these observations, our study identified BMI, blood pressure, CRP, and renal insufficiency as factors associated with DR, and highlighted immune–inflammatory/metabolic pathways and candidate druggable nodes (e.g., LY9, WWP2, ARHGAP1) that may enable cross-complication stratification and intervention. Future work in prospective multi-endpoint cohorts and genetic causal frameworks should map the mechanistic chains linking “molecular targets → modifiable risk factors (blood pressure, inflammation, nutritional status) → multi-organ complications,” and evaluate whether combined metabolic/immune-nutritional interventions can improve retinal and extra-retinal outcomes.

### Study strengths and limitations

This study has several notable strengths. We combined large-scale two-sample MR of 16,989 cis-eQTL genes and 2,923 cis-pQTL proteins with FinnGen DR subtype outcomes, which improved discovery power and clinical specificity. Robustness was further ensured through SMR+HEIDI validation and sensitivity analyses, reducing potential confounding. By integrating NHANES complex-survey analyses with MR and adjusting for key covariates such as diabetes duration and glycemic control, we achieved cross-validation between epidemiological and genetic evidence. Molecular targets were validated using bulk and single-cell transcriptomics, coupled with immune-cell mapping and pathway enrichment, adding biological plausibility. We also built a reproducible machine-learning model that showed strong external validity (AUC = 0.870), demonstrating translational potential. Finally, two-step mediation MR revealed mechanistic chains linking targets, risk factors (BMI/CRP/HBP), and DR subtypes, highlighting actionable genes such as LY9, WWP2, and ARHGAP1.

Nevertheless, several limitations should be noted. Reliance on cross-sectional data restricts causal inference, and part of the transcriptomic data was derived from peripheral blood rather than retinal tissue, which may not fully capture the retinal microenvironment. Future studies integrating retinal-specific omics, prospective cohorts, and animal experiments are needed to validate these findings and clarify mechanisms. With the advancement of big data and artificial intelligence, integrating multi-omics with machine learning may provide more precise tools for DR risk prediction and targeted intervention.

## Conclusion

This study, through systematic Mendelian randomization analysis, transcriptomic data mining, and mediation effect models, has delved into the potential pathogenic mechanisms of DR. We found that key risk factors such as BMI and HBP, under the regulatory effect of DR-specific targets such as LY9 and WWP2, indirectly influence the onset of DR through mediation effects. Furthermore, the study identified several potential targets associated with DR, such as LY9, WWP2, and ARHGAP1, which may jointly contribute to the progression of DR through immune-metabolic pathways. This study provides new biomarkers and targets for the early screening, precision medicine, and personalized treatment of DR, opening up new directions for future DR prevention and treatment strategies.

## Electronic supplementary material

Below is the link to the electronic supplementary material.

Supplementary material 1

## Data Availability

Publicly available datasets were analyzed in this study. The datasets supporting the conclusions of this article are included within the supplementary file.

## Abbreviations

AUC: Area under the curve
ANOVA: One-way analysis of variance
BUN: Blood urea nitrogen
CHD: Coronary heart disease
cis-eQTL: cis-expression quantitative trait loci
cis-pQTL: cis-protein quantitative trait loci
CI: Confidence interval
Cr: Creatinine
CRP: C-reactive protein
DBP: Diastolic blood pressure
DEG: Differentially expressed gene
DR: Diabetic retinopathy
DR-BKG: Diabetic background retinopathy
DR-BKG-SEVERE: Severe diabetic background retinopathy
DR-PROLIF: Proliferative DR
DR-NAS: Non-annotated DR
FDR: False discovery rate
GEO: Gene Expression Omnibus
GLM: Generalized linear model
GLU: Blood glucose
GO: Gene ontology
GWAS: Genome-wide association study
HbA1c: Glycated hemoglobin
HBP: Hypertension
HDL-c: High-density lipoprotein cholesterol
IV: Instrumental variable
HEIDI: Heterogeneity in dependent instruments
IVW: Inverse-variance weighted
KEGG: Kyoto encyclopedia of genes and genomes
LD: Linkage disequilibrium
LDL-c: Low-density lipoprotein cholesterol
MR: Mendelian randomization
MR-PRESSO: MR pleiotropy residual sum and outlier
NHANES: National Health and Nutrition Examination Survey
NK: Natural killer
OR: Odds ratio
RF: Random forest model
ROC: Receiver operating characteristic curves
SBP: Systolic blood pressure
scRNA-seq: Single-cell RNA sequencing
SE: Standard error
SDETG: Significant Differentially Expressed Treatment Gene
SNP: Single nucleotide polymorphism
SMR: Summary-data-based Mendelian randomization
ssGSEA: Single-sample gene set enrichment analysis
SVM: Support vector machine model
TC: Total cholesterol
TG: Triglyceride
UKB-PPP: UK Biobank Pharma Proteomics Project
UMAP: Uniform manifold approximation and projection
XGB: Extreme gradient boosting model
